# Historical Cohort Study of Congenital Isolated Hypoganglionosis of the Intestine: Determining the Best Surgical Interventions

**DOI:** 10.3390/biom13101560

**Published:** 2023-10-23

**Authors:** Yohei Yamada, Teizaburo Mori, Nobuhiro Takahashi, Takumi Fujimura, Motohiro Kano, Mototoshi Kato, Masataka Takahashi, Naoki Shimojima, Toshihiko Watanabe, Takako Yoshioka, Yutaka Kanamori, Tatsuo Kuroda, Akihiro Fujino

**Affiliations:** 1 Department of Pediatric Surgery, Keio University School of Medicine, Tokyo 160-8582, Japan; tkhsnbhr430@keio.jp (N.T.); mototoshi77@keio.jp (M.K.); a.fujino@keio.jp (A.F.); 2 Department of Pediatric Surgery, National Center for Child Health and Development, Tokyo 157-8535, Japan; mori.tei.pedsurg@gmail.com (T.M.); knmthr@keio.jp (M.K.); masataka.takahashi@med.toho-u.ac.jp (M.T.); kanamori-y@ncchd.go.jp (Y.K.); 3 Department of Pediatric Surgery, National Hospital Organization Saitama National Hospital, Saitama 351-0102, Japan; fujitaku3@gmail.com; 4 Department of Surgery, Tokyo Metropolitan Children’s Medical Center, Tokyo 183-8561, Japan; naoki.shimojima@gmail.com; 5 Department of Pediatric Surgery, Tokai University School of Medicine, Kanagawa 259-1193, Japan; watanabe-ts@tsc.u-tokai.ac.jp; 6 Department of Pathology, National Center for Child Health and Development, Tokyo 157-8535, Japan; yoshioka-t@ncchd.go.jp; 7 Kanagawa Children’s Medical Center, Yokohama 232-0066, Japan; kuroda-t@z8.keio.jp

**Keywords:** hypoganglionosis, myenteric plexus, Hu C/D, intestinal failure, stoma revision, small intestinal length, intestinal transplantation

## Abstract

No standard diagnostic method or surgical treatment for congenital isolated hypoganglionosis (CIHG) has been established. This study aimed to analyze the clinical outcomes of patients with CIHG and identify the best surgical interventions provided thus far. Data on surgical interventions in 19 patients were collected between 1992 and 2020, including the type of enterostomy, type of revision, and length of the intestines. Ganglion cells in the myenteric plexus were enumerated using Hu C/D staining. The ratio of the length of the small intestine to its height was defined as the intestinal ratio (IR). The outcomes were assessed using the stoma output, growth parameters including the body mass index (BMI), and parenteral nutrition (PN) dependency. All patients required a diverting enterostomy. The IR ranged from 0.51 to 1.75 after multiple non-transplant surgeries. The stoma types were tube-stoma, end-stoma, Santulli-type, and Bishop–Koop (BK)-type. Patients with Santulli- or BK-type stomas had better BMIs and less PN dependency in terms of volume than those with end-stomas or tube-stomas. Two patients with BK-type stomas were off PN, and three who underwent an intestinal transplantation (Itx) achieved enteral autonomy. The management of CIHG involves a precise diagnosis using Hu C/D staining, neonatal enterostomy, and stoma revision using the adjusted IR and Itx if other treatments do not enable enteral autonomy.

## 1. Introduction

Congenital isolated hypoganglionosis (CIHG) of the intestine is a disease wherein ganglion cells are present in the myenteric plexus but are small and scarce [1,2,3]; patients with CIHG display functional bowel obstruction during the neonatal period. Patients also have abdominal distention, vomiting, and abdominal pain from the neonatal period, and most require life-long parenteral nutrition (PN) and enterostomy to prevent enteritis due to bowel dysmotility. Although the survival prognosis of patients with CIHG has improved, their functional prognosis remains unsatisfactory.

The diagnostic criteria for CIHG are neonatal onset of bowel obstruction, hypoplastic myenteric plexus, and fewer ganglion cells in the affected intestine than in the normal intestine. Since no international consensus exists regarding the diagnostic criteria [4], CIHG has been confused with intestinal pseudo-obstruction [5,6], and few studies have solely analyzed patients with CIHG. The associated symptoms and histological findings are also similar to those observed in the ganglionic transition zone in Hirschsprung disease [7]; hence, CIHG has often been grouped as “variant Hirschsprung disease [8,9]” or “Hirschsprung disease-related allied disorder [2]”. Currently, growing evidence corroborates the disease entity as “CIHG”.

In 2012, Taguchi et al. reported a new classification of hypoganglionosis based on a precise histological examination utilizing quantitative morphometric measures [10]. A national survey conducted in Japan (161 institutes) identified 90 cases of CIHG over 10 years (2000–2009) on the basis of clinical symptoms and conventional hematoxylin–eosin and acetylcholine esterase staining [11]. Data on initial surgical treatment with short-term outcomes were also collected, and the findings suggested the benefit of creating an early jejunostomy [12,13]. More recently, studies have reported the usefulness of immunostaining with Hu C/D for counting ganglion cells [14,15]. In 2021, a detailed analysis of surgical specimens using Hu C/D immunostaining from 12 patients was reported in the US, and the authors named the disease entity “congenital myenteric hypoganglionosis”, because the abnormality was confined to the myenteric plexus [16]. However, considering the rarity of CIHG, little is known about its severity, treatment strategy after initial diverting enterostomy, and long-term outcomes.

In this study, we analyzed the clinical data from 19 patients from two centers who were pathologically confirmed as having CIHG by using Hu C/D staining of surgically resected specimens. This study included a large number of patients with histologically confirmed CIHG undergoing long-term follow-up; we aimed to identify the best surgical interventions based on the clinical parameters relevant to long-term outcomes. In the study, we also discussed the indications and technical points for intestinal transplantation (Itx).

## 2. Materials and Methods

### 2.1. Patients

Nineteen patients with histologically diagnosed CIHG were included in this study. They were followed up at Keio University Hospital and the National Center for Child Health and Development between 1992 and 2020. Patient data, including data on surgical history, stoma output, growth parameters, blood chemistry, and PN status, were retrospectively collected from the patients’ medical charts. This study was approved by the ethical committee of Keio University School of Medicine (approval number #20200047) and the National Center for Child Health and Development (approval number 2021-195).

### 2.2. Inclusion Criteria and Exclusion Criteria

All patients were diagnosed as having CIHG when they met the following criteria: (1) full-thickness biopsy findings were compatible with those of CIHG and (2) neonatal onset of ileus and persisting symptoms, which enabled distinction from immaturity of the ganglia. CIHG was defined as a reduced number of ganglion cells in the myenteric plexus, a reduced plexus area, and a reduced plexus length based on the histology, for which the details are described in the histological analysis section. The exclusion criteria encompass the unavailability of histological samples.

### 2.3. Histological Analysis

The specimen resected during the laparotomy was analyzed histologically using conventional hematoxylin–eosin staining and immunohistochemical staining with Hu C/D. Paraffin-embedded specimens were processed at a thickness of 3 μm and oriented vertically to the layers of the intestine for pathological analysis. The immunohistochemical study was conducted using the autostainer (Nichirei Histostainer 48A). The primary antibody used was the anti-HuC/HuD neuronal protein (mouse IgG2b monoclonal, clone 16A11; diluted to 1:200; Life Technologies, Carlsbad, CA, USA). Immunoperoxidase procedures were carried out using the streptavidin–biotin peroxidase method (Histofine; Nichirei Biosciences, Tokyo, Japan).

The counts and distribution of ganglion cells in the myenteric plexus in the jejunum, ileum, and colon were assessed by experienced pathologists according to previously reported criteria [15]. Briefly, immunostained neuronal cell bodies were quantified along a contiguous length of the myenteric plexus, which measured at least 10 linear millimeters. A ganglion cell body was only counted if the Hu C/D staining covered the nucleus across at least 50% of the nuclear circumference, and at least some cytoplasmic granular brown staining had to be present.

### 2.4. Measurement of the Intestinal Length, Intestinal Ratio, Stoma Output, and PN Depnedency

Bowel length was manually measured intraoperatively in situ along the antimesenteric border using a silk suture or a measuring tape by a single surgeon for each operation. Since the intestinal length increased according to the height, the ratio of the small intestinal length to height was recorded and defined as the intestinal ratio (IR). The stoma output was calculated as the average amount over a 5-day period, while the PN dependency was determined as the mean over a 7-day period when each patient was in a stable condition with their regular oral intake. These calculations were performed at intervals of at least 3 months following the last surgical interventions for each patient. PN dependency, evaluated as a percentage of the estimated required daily calorie or volume intake, was measured simultaneously with the measurement of the stoma output.

### 2.5. Statistical Analysis

The statistical analysis was performed using the non-parametric Mann–Whitney U test for numerical values and the chi-square test was performed for categorical analyses with GraphPad Prism 7.0 (GraphPad Software, Inc., San Diego, CA, USA). A *p* value < 0.05 was considered to indicate statistical significance. Correlation analyses were conducted using simple linear r regression models; unadjusted values are reported.

## 3. Results

### 3.1. Patient Demographics (Table 1)

**Figure 1:**
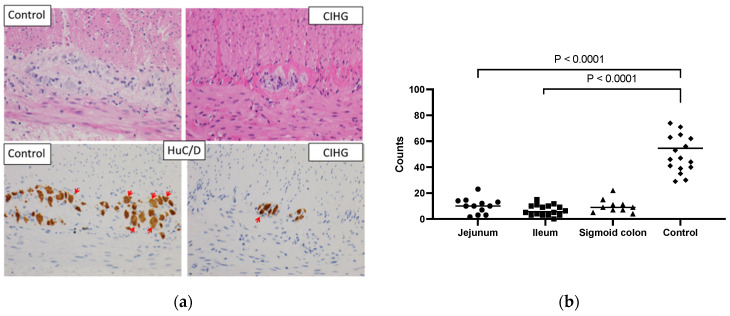
(**a**): The representative histological findings of HE and Hu C/D staining (at high power field (×400)). Red arrows indicate Hu C/D positive ganglion cells. The samples were obtained from a 4-month-old male patient diagnosed with CIHG (jejunum) and a 4-month-old male control patient (jejunum). A ganglion cell body was only counted if the Hu C/D staining covered the nucleus across at least 50% of the nuclear circumference. (**b**): The enumerated count of Hu C/D-positive ganglion cells (/cm) in resected specimens of CIHG patients and age-matched controls is shown. Statistical significance was observed in the numbers of Hu C/D-positive cells in both jejunal and ileal specimens of CIHG when compared to those in age-matched control specimens of either the jejunum or ileum.

The study included twelve female and seven male patients. At the time of the study, the patients’ ages at follow-up ranged from two to twenty-nine years. Since 2010, all patients newly diagnosed with CIHG have survived.Most patients were born at term later than 37 weeks, except for Pt 9 who was born at 35 weeks. Low birth weights were observed in two patients (Pt 5 and Pt 9). None of the patients had a family history of intestinal motility disorders. The counts of Hu C/D-positive cells per centimeter were consistently below 30 cm. The representative histological findings of HE and Hu C/D staining are shown (Figure 1a). The median enumerated counts of Hu C/D-positive ganglion cells were 10.0 cm (3.0–14.0: 95% CI of median) in the jejunum, 5.7 cm (3.0–10.0) in the ileum, 9.0 cm (5.0–15.0) in the sigmoid colon, and 54.5 cm (41.0–71.0) in the age-matched controls in the jejunum and the ileum, as shown in Figure 1b. Age-matched control specimens were obtained from patients with focal intestinal perforation, intestinal atresia, and ileus. Statistical significance was observed in the counts of Hu C/D positive cells between samples from CIHG and those from age-matched controls of either the jejunum or ileum. The examination of tissue samples focused specifically on the myenteric plexus, revealing significant observations such as a notable presence of diminutive ganglia, a surplus of cytologically immature ganglion cells, and a scarcity of neuropil. The extent of these alterations varied among the cases studied, but when compared to individuals of similar age in the control group, they were significantly more pronounced. The extent of hypoganglionosis was determined based on histological findings from either jejunal or ileal samples, or both. Consequently, all patients in this cohort exhibited myenteric hypoganglionosis to varying degrees in the small intestine. In some patients, step section analyses were also conducted using resected specimens to assess the longitudinal distribution of ganglion cells. The representative longitudinal distribution of ganglion cells in a resected specimen is shown in Figure S1.

**Table 1:** Patient demographics.

	Sex	Current Age	Birth History (Weight at Birth: g)	Day of Initial Surgery and the Procedure	Stoma Site at the Initial Surgery, Length (cm) from the LT and * a Percentage of the Total Estimated Small Intestinal Length	Hu C/D Count Jejunum /cm	Hu C/D Count Ileum /cm	Hu C/D Count Colon /cm
1	M	29	Term 39 (3484)	Day 2, Ileostomy	160 cm (89.8%)	12	5	15
2	M	25	Term 37 (3012)	Day 3, Ileostomy	NA	10	NA	4
3	M	25	Term 39 (3452)	2 months, Jejunostomy	60 cm (33.8%)	3	11	11
4	F	20	Term 39 (2788)	1 month, Ileostomy	30 cm from TI (81.1%)	NA	3	9
5	F	18	Term 37 (2368)	Day 2, Jejunostomy	70 cm (47.9%)	NA	9	7
6	M	17	Term 40 (3480)	Day 2, Jejunostomy	75 cm (42.1%)	1.6	NA	NA
7	F	14	Term 41 (2966)	Day 1, Ileostomy	NA	NA	6.4	NA
8	F	12	Term 37 (2832)	1 month, Ileostomy	45 cm from TI (71.9%)	NA	10	7
9	M	12	Preterm 35 (1828)	Day 16, Gastrostomy	0 cm (0%)	14	3	9
10	F	12	Term 40 (3408)	Day 2, Ileostomy	40 cm from TI (77.3%)	NA	4	NA
11	M	11	Term 40 (3380)	Day 0, Ileostomy	5 cm from TI (97.1%)	NA	9	NA
12	F	10	Term 38 (3084)	Day 1, Jejunostomy	30 cm (17.9%)	10	5	NA
13	F	9	Term 37 (2625)	Day 3, Ileostomy	115 cm (74.6%)	7	15	NA
14	F	7	Term 39 (3456)	Day 1, Jejunostomy	70 cm (39.5%)	3	3	NA
15	M	7	Term 37 (2917)	Day 20, Jejunostomy (Full thickness biopsy at day 2)	45 cm (27.7%)	13	NA	NA
16	F	5	Term 38 (3340)	Day 0, Jejunostomy	70 cm (40.2%)	10	0	5
17	F	3	Term 39 (3240)	Day 30, Jejunostomy (Full thickness biopsy at day 3)	50 cm (29.1%)	14.5	11.7	11.2
18	F	3	Term 40 (3334)	Day 8, Jejunostomy (Full thickness biopsy at day 2)	40 cm (23%)	NA	10	NA
19	F	2	Term 38 (2836)	Day 19, Jejunostomy	40 cm (25%)	23	3	22

NA: not available, LT: ligament of Treitz, TI: terminal ileum. * A percentage representing the length of the small intestine to each stoma, relative to the total estimated length of the small intestine. The estimated small bowel length was obtained by the following formula: small bowel length = 2.736 × BW^0.512^ [17].

### 3.2. Initial Diverting Enterostomy

All patients developed ileus and required gastrostomy or enterostomy during the neonatal period (Table 1). In this study, the small intestinal length to the stoma site was manually measured intraoperatively in situ along the antimesenteric border using a silk suture or a measuring tape by a single surgeon. A stoma positioned proximal to the 50% mark of the estimated total small bowel was categorized as a jejunostomy, while one positioned distal to the 50% mark was categorized as an ileostomy. Other stomas created in the later stages of life were defined as enterostomies with approximate lengths from the LT or TI, which are indicated in Table S1.

Theinitial surgeries were performed between 0 and 60 days. Some patients underwent a full-thickness biopsy only during the first laparotomy. The first enterostomy was performed after a median duration of 10.5 days (11 patients underwent stoma creation within 3 days after birth). Among the patients, eight underwent an ileostomy, ten underwent a jejunostomy, and one underwent a jejunostomy following gastrostomy. The length of the small intestine to the stoma site ranged from 30 to 160 cm.

Of note, 11 had to undergo intestinal resection and another enterostomy proximal to the existing stoma site because of the ineffective drainage of dilated bowel loops. In our centers, full-thickness biopsy specimens have been sampled from the stoma site, ileum, and sigmoid colon if CIHG is suspected intraoperatively since 2010. Notably, as most of the patients were referred to our centers after undergoing the initial surgical interventions, the data on the initial full-thickness biopsies were unavailable for all patients.

### 3.3. Type of Stoma, Residual Intestinal Length, and Distal Colon (Table 2 and Figure 2)

**Figure 2:**
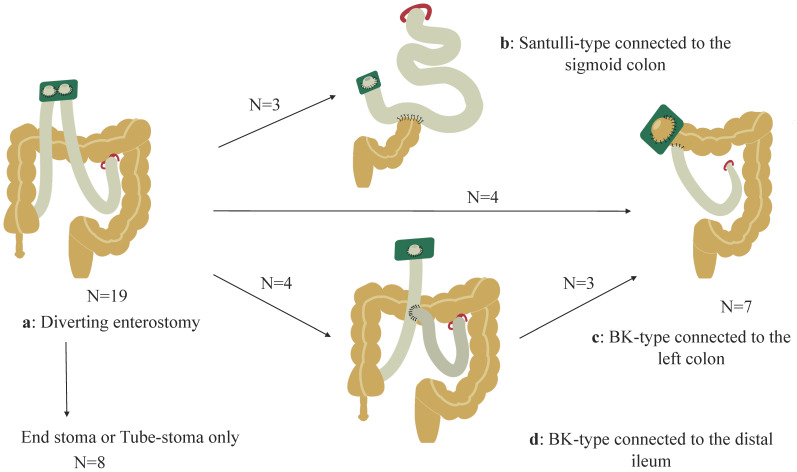
Non-transplant surgical procedures are described. (**a**): Initial diverting stoma. Among the patients, eight retained either an end-stoma or tube-stoma, three had Santulli-type stomas connected to the sigmoid colon (**b**), and four had Bishop–Koop (BK)-type stomas connected to the left half of the transverse colon (**c**). Moreover, four patients initially had ileal connections, (**d**) and subsequently, three of the four patients underwent a stoma revision involving a connection with the left half of the transverse colon (**c**).

Various non-transplant surgical interventions were performed after the initial enterostomy (Figure 2a). Intestinal resection was primarily performed as a result of recurrent enteritis or the inability to consume food orally due to inadequate bowel decompression. Conversely, stoma revisions, such as Santulli-type stoma or Bishop–Koop (BK)-type stomas, aimed to rehabilitate the distal colon and decrease the stoma output. Details are described in Table S1, and the current status is summarized in Table 2. In this study, the ratio of the intraoperatively measured small intestinal length (cm) to the height (cm) was defined as the IR. For example, when the residual small intestinal length was 80 cm from the LT and the height was 100 cm, the corresponding IR was 0.8. Table 2 shows the timing of the most recent surgical interventions, IR, type of stoma, residual distal colon, stoma output (mL/kg/day), PN dependency assessed by a percentage of the total estimated required calories or volume per day, height (Z-score), body weight (Z-score), BW to height ratio, and body mass index (BMI; Z-score) of each patient. Two patients (Pt 1 and Pt 6) received tube-stomas after several bowel resections, resulting in IRs of 0.51 and 0.58, respectively. Six patients received endo-enterostomas with residual intestinal lengths indicated by IRs ranging from 0.60 to 1.65. Three patients received Santulli-type stomas, with IRs of 0.81, 0.96, and 1.27, and the sigmoid colon was connected distally to the Santulli-type stoma (Figure 2b). Eight patients received BK-type stomas with IRs ranging from 0.51 to 1.13. The left half of the transverse colon was connected distally to the BK-type stoma in four patients (Figure 2c), and the ileum was connected to the total colon in four patients (Figure 2d). Moreover, three of the four patients with BK-type stomas with an ileal connection subsequently underwent ileal resection with a right hemicolectomy (this resulted in a total of seven patients having the status shown in Figure 2c). Notably, the pre-iTx data were utilized in four patients (Pt 1, Pt 2, Pt 3, and Pt 6) who subsequently underwent iTx.

**Table 2:** Current status of the CIHG patients.

	IR = Small Intestine (cm)/Height (cm), Measured at the Latest Surgery	Stoma Type	Distal Colon	Stoma Output mL/kg/day	PN Dependency % of the Total Estimated Calories	PN Dependency % of the Total Estimated Volume	Height Z Score	BW Z Score	BW /Height	BMI Z Score
1	76/147.4 = 0.51 (14 years old) *Measured at iTx	Tube enterostoma	T/C	75 *before iTx	103.2	243.4	−2.5	−1.3	0.27	−0.4
2	102/156 = 0.65 (15 years old) *Measured at iTx	End stoma	T/C	30.6 *before iTx	51.3	116.1	−1.8	−2.4	0.21	−4.3
3	107/103 = 1.03 (11 years old) *Measured at iTx	End stoma	T/C	25.5 *before iTx	42.8	93.3	−6.0	−2.6	0.15	−1.7
4	120/148 = 0.81 (12 years old)	Santulli	S/C	28.8	24	45.2	−0.9	0.4	0.37	1.0
5	160/126 = 1.27 (9 years old)	Santulli	S/C	28.8	35.2	98.6	−0.8	−0.9	0.3	−0.6
6	75/128.5 = 0.58 (10 years old) *Measured at iTx	Tube enterostoma	Rectum	107.2 *before iTx	70.8	195.9	−1.1	−1.6	0.16	−3.3
7	90/135.9 = 0.66 (12 years old)	BK	T/C	46.4	29.4	81.4	0.1	−0.4	0.25	−0.5
8	115/92.5 = 1.24 (4 years old)	End stoma	T/C	31.2	67.4	63.8	−2.0	−1.4	0.23	−1.0
9	63/65.5 = 0.96 (1.5 years old)	Santulli	S/C	44.3	56.2	68.5	−4.3	−2.6	0.16	−3.3
10	160/96.8 = 1.65 (4 years old)	End stoma	Total colon	38.7	4.8	13.3	−3.3	−2.1	0.2	−1.9
11	75/125 = 0.6 (8 years old)	End stoma	T/C	52	21.8	93.7	−0.1	−1.1	0.18	−2.2
12	100/93 = 1.075 (3 years old)	BK	T/C	32.9	0	0	−1.3	−0.8	0.17	−0.0
13	90/104.8 = 0.86 (8 years old)	End stoma	S/C	124.8	63.2	187.2	−3.9	−2.2	0.15	−1.7
14	100/88.3 = 1.13 (3 years old)	BK	T/C	26.9	54.6	67	−3.0	−2.3	0.13	−2.1
15	55/67 = 0.82 (2 years old)	BK	T/C	51.1	0	0	−2.1	−1.1	0.18	0.1
16	65/85 = 0.76 (3 years old)	BK	Ileum + total colon	78.4	55.8	84.4	−1.6	−1.6	0.14	−1.4
17	75/88 = 0.85 (3 years old)	BK	T/C	46.5	24.4	43.7	−0.6	−0.4	0.14	0.0
18	50/66 = 0.75 (10 months old)	BK	T/C	57.9	39	69.6	−3.5	−2.7	0.12	−1.0
19	40/78.9 = 0.51 (2 years old)	BK	T/C	93	40.9	73	−1.8	−2.0	0.12	−1.3

* These values were obtained before iTx.

### 3.4. Associations among the Intestinal Length, Stoma Revision, PN Dependency, and Growth Parameters

**Figure 3:**
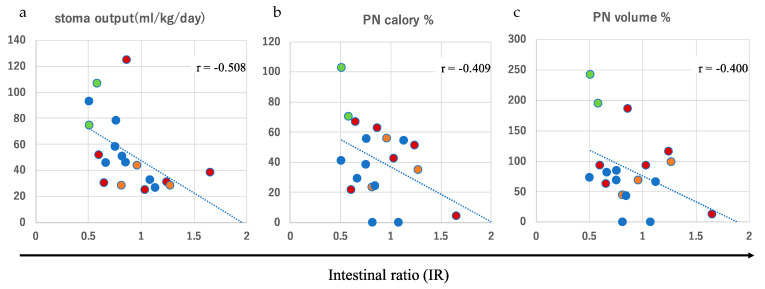
Moderate and weak linear correlations between the intestinal ratio (IR) and stoma output (r = −0.508) (**a**), parenteral nutrition (PN) based on calories (r = −0.409) (**b**), and PN based on volume (r = −0.400) (**c**). Red circle: end-stoma; green circle: tube-stoma; blue circle: Bishop–Koop (BK)-type stoma; orange circle: Santulli-type stoma.

To quantitatively assess the absorptive capacity of the hypoganglionic intestine, the average stoma output and IR were plotted (Figure 3). The stoma output was calculated as the mean amount over 5 days when each patient was in a stable condition with their usual oral intake, and the measurements were performed at intervals of at least 3 months after the last surgical interventions. A moderate linear correlation was observed between the stoma output and the IR in all patients with a correlation coefficient of r = −0.508 (*p* = 0.0261) (Figure 3a). In addition, a weak linear correlation was observed between the IR and PN dependency, evaluated as a percentage of the estimated required daily calorie intake (r = −0.409, *p* = 0.0818) and volume intake (r = −0.400, *p* = 0.0934), respectively (Figure 3b,c). These values were obtained simultaneously with the measurement of the stoma output. These findings suggest that the IR may allow us to predict the stoma output and PN dependency in patients with CIHG.

**Figure 4:**
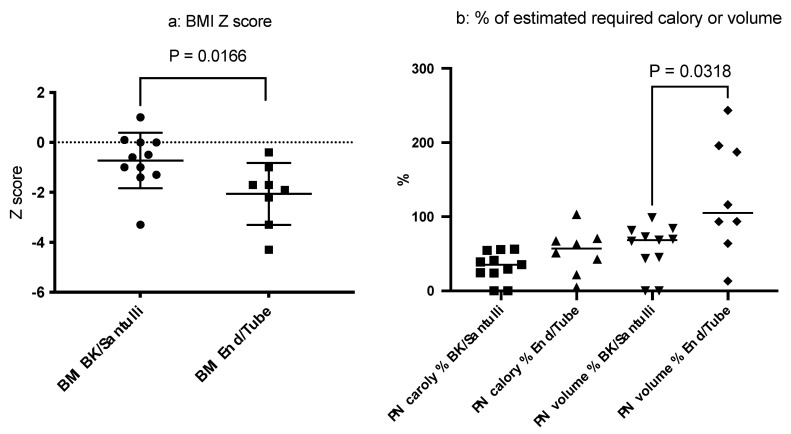
Comparison of the body mass index (BMI) (**a**) and parenteral nutrition (PN) dependency (**b**) based on the stoma type. Patients with Santulli- or BK-type stomas had better BMIs than those with end-stomas or tube-stomas only (*p* = 0.0166). Additionally, a trend for less PN dependency based on volume was observed in the revision group (*p* = 0.0318).

To investigate the clinical effect of stoma revision, either of the Santulli- or the BK-type, we compared the background characteristics (current age, age at intervention, sex, and the IR) and outcomes of stoma revision according to the stoma output, PN dependency, and growth parameters between the group that underwent revision (either Santulli- or BK-type) and the group with an end-stoma or tube-stoma only (Table 3). Although no statistical differences were observed in the IR or the average stoma output between the two groups, the patients in the revision group showed a higher BMI as assessed using Z-scores at follow-up (*p* = 0.0192) than those in the group with an end-stoma or tube-stoma only (Figure 4a). In addition, a tendency toward a slight PN dependency (*p* = 0.0318) in volume (Figure 4b) was observed in the group that underwent revision than in the group with an end-stoma or tube-stoma only. These findings indicate that stoma revision involving distal colon rehabilitation may independently affect the PN volume dependency and physical growth, as assessed using the BMI. Indeed, two patients with a BK-type stoma revision were PN-free and remained PN-free for 3 and 8 years after the revision, respectively. Collectively, these findings suggest that the IR and type of revision are both important determinants of the stoma output, PN dependency according to volume, and physical growth in patients with CIHG.

### 3.5. iTx

Although two patients who underwent a BK-type stoma revision achieved independence from PN support, most of the other patients, regardless of stoma type, were still dependent on PN support and all retained enterostomas at follow-up. In this study, four patients were scheduled for iTx, mainly because of their severely impaired quality of life and absence of accessible central veins for PN.

Four patients (Pt 1, Pt 2, Pt 3, and Pt 6) underwent living-donor iTx at ages 10, 11, 14, and 15 years, respectively. Before iTx, various non-transplant surgical interventions were performed for all of these patients, as described above. The median number of non-transplant interventions was 5.5, and none of these were effective for achieving enteral autonomy. The details of the iTxs are summarized in Table 4. All donor sources were living-related, and the intestinal graft was resected 15 cm proximal from the Bauhin valve (preserved for the live donors), and the length of the graft was one-third of the total intestine (110–150 cm). The residual length of the native intestine with dysmotility proximal to the graft ranged from 5 to 30 cm from the LT, which was determined on the basis of the preoperative upper gastrointestinal examination and the intraoperative vascular supply. One patient (Pt 2) lost the graft because of chronic rejection 2 years after iTx. Complete stoma reversal was achieved at postoperative year 3 (POY 3) in Pt 1, POY 11 in Pt 3, and POY 2 in Pt 6 after iTx. Regarding the native intestine distal to the graft, the sigmoid colon was anastomosed to the graft in Pt 1 and Pt 3, and the part of the ileum anastomosed to the rectum was utilized in Pt 6. Despite the limited length of the graft and the native distal bowel with functional dysmotility, reversal of the stoma allowed freedom from PN support in all three patients. All three patients who retained the functioning graft had a normal diet and 6–7 bowel movements per day and showed growth catch-up. They are currently undergoing endoscopic surveillance every 4–6 months.

## 4. Discussion

In this study, we analyzed the clinical details of 19 patients with histologically confirmed CIHG, who were followed up for 2–29 years (mean follow-up = 12.8 years). During the study period before 2010, one patient was suspected to have CIHG. However, no histological details could be retrieved for this patient. As a result, our analysis was conducted on the data from 19 patients who were alive during this period. The survival rate in the current cohort has been 100% since 2010, which is better than the 78% obtained in a national survey conducted in 2009 [12]. However, since most patients are PN-dependent with enterostomy over the long term, the functional outcomes and quality of life of these patients are still far from satisfactory.

First and most importantly, a precise and early diagnosis of CIHG should be made to provide timely surgical intervention. To achieve this goal, obtaining adequate surgical samples is of paramount importance. If CIHG is suspected preoperatively or intraoperatively and Hirschsprung disease is ruled out based on the presence of ganglion cells, the guidelines established in Japan suggest multiple full-thickness biopsy specimens, including those of the sigmoid colon, ileum, and jejunum, be collected when the initial stoma is created [18]. Additionally, immunohistochemical staining with Hu C/D helps to quantify ganglion cells; the number of ganglion cells in the normal intestine obtained from age-matched control samples ranged from 41.0 to 71.0 cm, while that in CIHG was below 20–30 cm [14,16]. It is worth noting that our quantified ganglion cell values differ from those reported by other researchers (Taguchi [11], Yoshimaru [14], Kapur [15,16], Graham [19]. One significant contributing factor to this variation is the differences in our methodological approach. Specifically, we utilized a different autostainer, clones of antibodies, and concentrations of antibodies to identify and quantify Hu C/D-positive cells in our study.

Our cell-counting criteria were established following the guidelines outlined in Swaminathan and Kapur [15], which were aimed at ensuring consistency and accuracy in our analysis. Additionally, we acknowledge that the size of the region evaluated can influence myenteric ganglia counts. However, as demonstrated in our current study, even a single slice of the circumferential sample still exhibited statistically significant differences in ganglion counts between normal intestines and CIHG cases. We believe that our method, when executed by experienced pathologists, is straightforward and pragmatic for diagnosing CIHG.

Given the rarity of CIHG, a central diagnostic system is being established in Japan by experienced pathologists. Whether the number and distribution of ganglion cells enumerated using Hu C/D staining affect the functional capacity of the intestine warrants further study in order to personalize treatment options. Additionally, regarding the investigation of mesenchymal components in the context of intestinal motility, including immunohistochemistry for SOX-10, CD56, nNOS, and Calretinine, we are actively conducting a comprehensive analysis. This study is ongoing and will be presented in a future publication.

Second, the importance of the initial upper jejunostomy should be emphasized. As previously reported by Watanabe et al. [13], 11 of 13 patients who initially underwent an ileostomy or non-upper jejunostomy subsequently required an upper jejunostomy to obtain a functional ostomy in the first several months after birth. Since the middle of 2010, the idea of an upper jejunostomy with a length of 50 cm or less from the LT during the neonatal period was incorporated into our CIHG treatment strategy and was applied to Pt 12, Pt 14, Pt 15, Pt 17, Pt 18, and Pt 19. Although a definitive intraoperative diagnosis of CIHG is often challenging in the absence of experienced pathologists, an approach that creates an ileostomy pending a review of permanent sections will be a judicious option.

Third, after functional enterostomies, subsequent non-transplant surgical interventions were divided into two groups: (1) end-stoma with a non-functional distal colon (n = 6) and tube-stoma only (n = 2) with a minimal distal colon, and (2) Santulli-type stoma revision with the distal colon (n = 3) and BK-type stoma revision with the distal colon (n = 8). Thereafter, the major questions arise regarding which type of stoma and how long the optimal small intestinal length should be to provide the most benefits to patients with CIHG. This discussion is complicated because each option has its advantages and disadvantages.

The standard small intestinal length was previously approximated with the height or the BW by using the following formula: Ln (small bowel length) = 6.741–−(80.409/height), small bowel length = 2.736 × BW^0.512^ [17]. Normally, the ratio of the small intestinal length to the height falls between 3 and 3.75 in the first 5 years of life. Before 2010, the adoption of various small intestinal lengths led to inconsistent functional outcomes. From 2010 onwards, as we succeeded in a series of cases in preventing severe enteritis and maintaining the oral intake by adjusting the IR to around 0.8–1.0, we advocated for the IR as one of the indicators of an optimal small intestinal length in CIHG. In addition, the linear approximation obtained from our cohort suggested that the estimated stoma output in patients with CIHG ranged between 40.4 mL/kg and 55.9 mL/kg if the IR ranged between 0.8 and 1.0, respectively.

With regard to the type of stoma, BK- or Santulli-type stomas may offer benefits over end stomas or tube-stomas only in growth parameters assessed using the BMI and PN dependency assessed based on the volume. Theoretically, if the distal colon is used in either type of stoma, it can be speculated that the absorptive capacity should be augmented. In addition, BK- or Santulli-type stoma revision allows for distal colon rehabilitation. However, in either type of stoma, if the remnant distal intestine is too long, it would greatly increase the risk of severe enteritis, as was observed in four patients (Pt 7, Pt 14, Pt 16, and Pt 19). In these patients, the ileum and total colon were utilized as the distal intestine in the BK-type stoma. Thus, our current strategy consists of an initial jejunostomy followed by either a Santulli- or BK-type stoma revision with an adjusted IR of around 0.8–1.0, which anastomosed to the short segment of the distal colon. Indeed, two patients achieved a PN-free status after BK-type stoma revision with IRs of 0.82 and 1.08, and the left side of the colon was connected distally to the BK-type stoma. Importantly, some patients underwent daily irrigation of the distal intestine from the stoma or the anus prior to stoma revision as a component of intestinal rehabilitation, which we believe is important to obtain successful adaptation after the revision. Although the optimal timing of stoma revision remains unclear, the good functional outcomes in Pt 12, Pt 14, Pt 15, Pt 17, and Pt 18 led us to speculate that early stoma revision may help with distal colon adaptation.

Fourth, four patients underwent living-donor iTx, and three of them have retained functional grafts, which provided them with freedom from PN and complete reversal of stoma for 7–15 years. The fact that only three patients with iTx, out of the 19 patients with CIHG in this study, achieved both a PN-free status and complete reversal of enterostomy, led us to suggest that iTx should be considered a practical option to obtain enteral autonomy in CIHG. The drawbacks of iTx are associated with poor long-term graft survival and the need for lifelong immunosuppression. Efforts to improve iTx outcomes are beyond the scope of the current study, but our current strategy to consider ITx holds true when half of the central vein access sites have been lost, and the quality of life has been severely impaired. Notably, intentionally leaving a certain length of the native proximal small intestine and native distal intestine to the graft during the transplant procedure did not incur any functional obstruction after iTx. Furthermore, by preserving a certain length of the native intestine, intestinal continuity can be restored even if the intestinal graft needs to be resected because of irreversible rejection, as seen in Pt 2.

Our study has several limitations. Only a small number of patients could be included, which limited the statistical analysis. CIHG is a rare condition that explains the limited number of patients and the retrospective design of the study. Moreover, the study covered a very long time frame, which might have induced a historical bias. Nevertheless, our findings provide practical information for managing patients with CIHG.

## 5. Conclusions

In conclusion, our current concept of treating CIHG consists of (1) precise diagnosis via thorough pathological assessment using Hu C/D immunostaining of full-thickness biopsy specimens, (2) initial jejunostomy in the neonatal period to obtain functional diversion, (3) either Santulli- or BK-type stoma revision utilizing an optimal length of the proximal and distal intestines, and (4) iTx if the above treatment fails to provide a sustainable quality of life.

## Figures and Tables

**Table 3:** Characteristics (a) and clinical outcomes (b) of the patients according to the stoma type.

a	Stoma Revision (Santulli or BK), n = 11 (Median)	End-Stoma or Tube-Stoma Only, n = 8 (Median)	*p* Value
Age	2–20 (7)	9–14 (11.5)	0.2791
Age at intervention	0.8–12 (3)	0.5–8 (4)	0.7037
Sex	Male: 2 Female: 9	Male: 5 Female: 3	0.0739
Intestinal ratio (IR)	0.51–1.27 (0.82)	0.51–1.65 (0.755)	0.7345
**b**	**Stoma Revision (Santulli or BK), n = 11, (Median)**	**End Stoma or Tube Stoma Only, n = 8 (Median)**	***p* Value**
Stoma output mL/kg	28.8–93 (46.4)	25.5–124.8 (45.35)	0.7002
Height Z score	−4.3–0.1 (−1.6)	−5.8–−0.7 (−2.25)	0.2816
BW Z score	−3.5–0.4 (−1.1)	−2.6–−1.3 (−2.2)	0.1704
BMI Z score	−3.3–1.0 (−0.6)	−4.3–−0.4 (−1.8)	0.0166
PN calories	0–56.2 (35.2)	4.8–103.2 (57.2)	0.1051
PN volume	0–98.6 (68.5)	13.3–243.4 (104.9)	0.0318

**Table 4:** Characteristics and clinical outcomes of the patients who underwent an intestinal transplantation (iTx). NA: not available.

Pt No	Sex	Age at iTx (Years)	Length of the Native Intestine Proximal to the Graft (cm)	Graft Length (cm)	Native Intestine Distal to the Graft	Status of Stoma	PN Dependency	Time since iTx (Years)	BMI Pre-iTx (Z Score)	BMI Post-iTx (Z Score)	Height Pre-iTx (Z Score)	Height Post-iTx (Z Score)
1	M	14	30	150	S/C	Reversed 3 years after iTx	Off for 13 years	15	−0.04	0.94	−3.55	−1.92
2	M	11	5	110	S/C	Reversed 11 years after iTx	Off for 3 years	14	−1.74	−1.46	−5.97	−4.2
3	M	15	26	150	NA	End stoma	Back to PN	Graftectomy 2 years after iTx due to chronic rejection	−4.28	NA	−2.19	NA
6	M	10	15	150	Ileum (30 cm) + Rectum	Reversed 2 years after iTx	Off for 5 years	7	−3.34	−2.57	−1.39	−0.86

## Data Availability

The data presented in this study are available on request from the corresponding author.

## Supplementary Materials

The following supporting information can be downloaded at: https://www.mdpi.com/article/10.3390/biom13101560/s1, Figure S1: Representative step section analyses in resected specimens from representative patients with CIHG to assess the longitudinal distribution of the ganglion cells. The numbers on the *X*-axis indicate the order of the specimens, ranging from the proximal intestine to the distal intestine. The *Y*-axis represents the number of ganglion cells per centimeter. Table S1: The details of the non-transplant surgical procedures for each patient.

## References

[1] Borchard F., Meier-Ruge W., Wiebecke B., Briner J., Müntefering H., Födisch H.F., Holschneider A.M., Schmidt A., Enck P., Stolte M. (1991). Disorders of the innervation of the large intestine--classification and diagnosis. Results of a consensus conference of the Society of Gastroenteropathology 1 December 1990 in Frankfurt/Main. Pathologe.

[2] Holschneider A.M., Meier-Ruge W., Ure B.M. (1994). Hirschsprung’s disease and allied disorders--a review. Eur. J. Pediatr. Surg..

[3] Dingemann J., Puri P. (2010). Isolated hypoganglionosis: Systematic review of a rare intestinal innervation defect. Pediatr. Surg. Int..

[4] Martucciello G., Pini Prato A., Puri P., Holschneider A.M., Meier-Ruge W., Jasonni V., Grosfeld J.L. (2005). Controversies concerning diagnostic guidelines for anomalies of the enteric nervous system: A report from the fourth International Symposium on Hirschsprung’s disease and related neurocristopathies. J. Pediatr. Surg..

[5] Rudolph C.D., Hyman P.E., Altschuler S.M., Christensen J., Colletti R.B., Cucchiara S., Carlo D.L., Alex F., Hillemeier A., McCallum R. (1997). Diagnosis and treatment of chronic intestinal pseudo-obstruction in children: Report of consensus workshop. J. Pediatr. Gastroenterol. Nutr..

[6] Thapar N., Saliakellis E., Benninga M.A., Borrelli O., Curry J., Faure C., De Giorgio R., Gupte G., Knowles C.H., Staiano A. (2018). Paediatric Intestinal Pseudo-obstruction: Evidence and Consensus-based Recommendations From an ESPGHAN-Led Expert Group. J. Pediatr. Gastroenterol. Nutr..

[7] Meier-Ruge W.A., Brunner L.A., Engert J., Heminghaus M., Holschneider A.M., Jordan P., Piket G., Posselt H.G., Schärli A. (1999). A correlative morphometric and clinical investigation of hypoganglionosis of the colon in children. Eur. J Pediatr. Surg..

[8] Puri P. (1997). Variant Hirschsprung’s disease. J. Pediatr. Surg..

[9] Puri P., Gosemann J.H. (2012). Variants of Hirschsprung disease. Semin. Pediatr. Surg..

[10] Taguchi T., Masumoto K., Ieiri S., Nakatsuji T., Akiyoshi J. (2006). New classification of hypoganglionosis: Congenital and acquired hypoganglionosis. J. Pediatr. Surg..

[11] Taguchi T., Ieiri S., Miyoshi K., Kohashi K., Oda Y., Kubota A., Watanabe Y., Matsufuji H., Fukuzawa M., Tomomasa T. (2017). The incidence and outcome of allied disorders of Hirschsprung’s disease in Japan: Results from a nationwide survey. Asian J. Surg..

[12] Watanabe Y., Kanamori Y., Uchida K., Taguchi T. (2013). Isolated hypoganglionosis: Results of a nationwide survey in Japan. Pediatr. Surg. Int..

[13] Watanabe Y., Sumida W., Takasu H., Oshima K., Kanamori Y., Uchida K., Taguchi T. (2015). Early jejunostomy creation in cases of isolated hypoganglionosis: Verification of our own experience based on a national survey. Surg. Today.

[14] Yoshimaru K., Taguchi T., Obata S., Takemoto J., Takahashi Y., Iwanaka T., Yanagi Y., Kuda M., Miyoshi K., Matsuura T. (2017). Immunostaining for Hu C/D and CD56 is useful for a definitive histopathological diagnosis of congenital and acquired isolated hypoganglionosis. Virchows Arch..

[15] Swaminathan M., Kapur R.P. (2010). Counting myenteric ganglion cells in histologic sections: An empirical approach. Hum. Pathol..

[16] Kapur R.P., Bellizzi A.M., Bond S., Chen H., Han J.S., LeGallo R.D., Midgen C., Poulin A.A., Uddin N., Warren M. (2021). Congenital Myenteric Hypoganglionosis. Am. J. Surg. Pathol..

[17] Struijs M.C., Diamond I.R., de Silva N., Wales P.W. (2009). Establishing norms for intestinal length in children. J. Pediatr. Surg..

[18] Muto M., Matsufuji H., Taguchi T., Tomomasa T., Nio M., Tamai H., Tamura M., Sago H., Toki A., Nosaka S. (2018). Japanese clinical practice guidelines for allied disorders of Hirschsprung’s disease, 2017. Pediatr. Int..

[19] Graham K.D., López S.H., Sengupta R., Shenoy A., Schneider S., Wright C.M., Feldman M., Furth E., Valdivieso F., Lemke A. (2020). Robust, 3-Dimensional Visualization of Human Colon Enteric Nervous System Without Tissue Sectioning. Gastroenterology.

