# Supplementary material for: Historical Cohort Study of Congenital Isolated Hypoganglionosis of the Intestine: Determining the Best Surgical Interventions
Source: Biomolecules. 2023 Oct 23;13(10):1560. doi: 10.3390/biom13101560 (PMC10605557; doi:10.3390/biom13101560)
Supplement: Supplementary file 1 [file biomolecules-13-01560-s001.zip › biomolecules-2546364-supplementary.pdf]

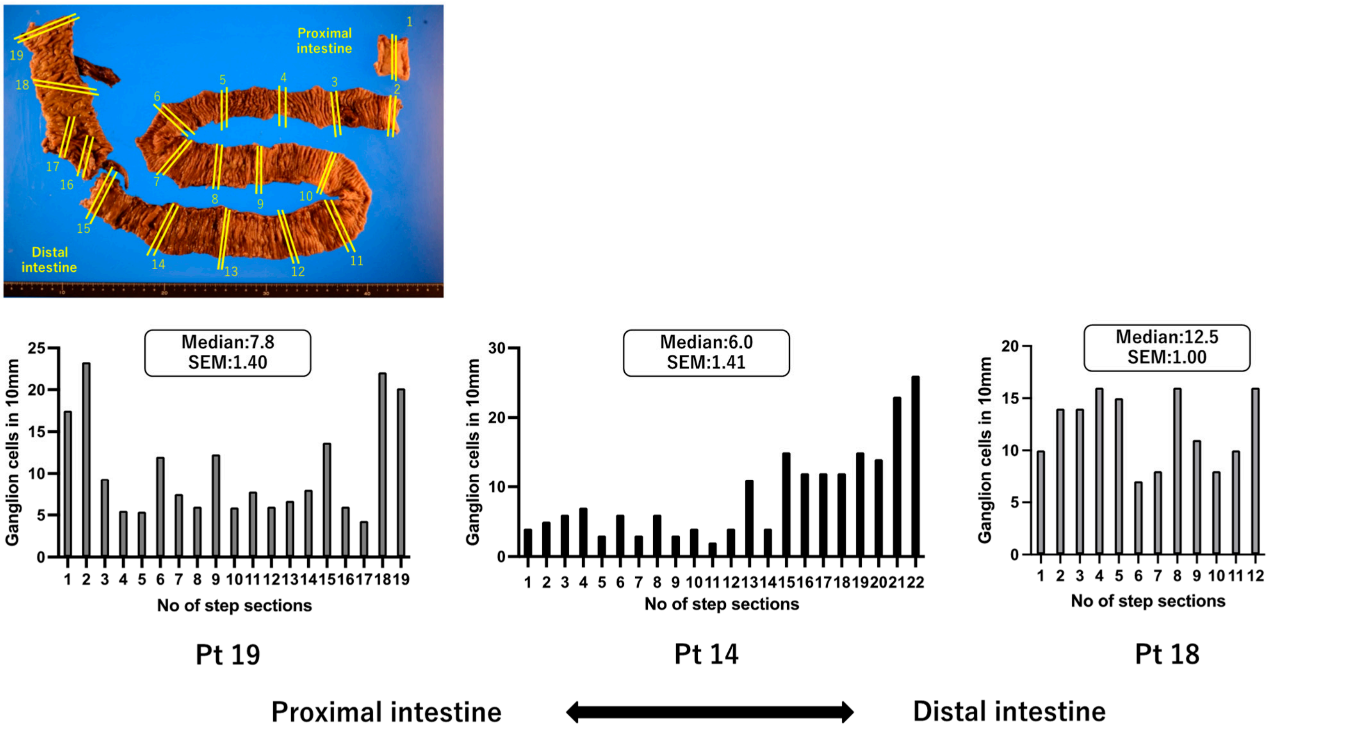

Figure S1. Representative step section analyses in resected specimens from representative patients with CIHG to assess longitudinal distribution of ganglion cells. The numbers on the X-axis indicate the order of the specimens, ranging from the proximal intestine to the distal intestine. The Y-axis represents the number of ganglion cells per centimeter.

Table S1. The details of non-transplant surgical procedures for each patient.

	Details of major surgical interventions, * length of small intestine is noted in brackets
1	Proximal enterostomy (85 cm) at the age of 1, enterostomy (20 cm) with distal ileal resection and right hemicolectomy at 4 years old, tube enterostomy at 5 years old. ITx at 14 years old (Residual length of small intestine was 76cm).
2	Proximal enterostomy (NA) at 14 days old, enterostomy (NA) with distal ileal resection and right hemicolectomy at 6 months, ITx at 15 years old (Residual length of small intestine was 102 cm).
3	Proximal tube enterostomy (70 cm) with distal ileal resection and right hemicolectomy at 1 year old, enterostomy (55 cm) at 2 years old, ITx at 11 years old (Residual length of small intestine was 107 cm).
4	Enterostomy (50 cm) with colon patch (10 cm) and subtotal colectomy at 3 years of age, Santulli stoma revision (120 cm) with S/C connection at 12 years old
5	Proximal enterostomy (NA), enterostomy (160 cm) with colon patch (10 cm) and distal ileal resection and subtotal colectomy at 9 year of age, Santulli stoma revision (160 cm) with S/C connection at 9 years old.

6	Distal ileal resection with total colectomy and jejunal rectal anastomosis at 2 years of age, tube enterostomy (75 cm) at 6 years old, ITx at 10 years old (Residual length of small intestine was 75 cm).
7	Proximal enterostomy (NA), Santulli stoma revision (NA) with ileal connection at 8 years old, BK stoma revision (90cm) with T/C connection at 12 years old
8	Proximal enterostomy (65 cm from the TI) at 3 years old, enterostomy (115 cm) with distal ileal resection and right hemicolectomy at 4 years old
9	Enterostomy (27 cm) at the age of 6 months, enterostomy (63 cm) with colon patch (10 cm) and distal ileal resection and subtotal colectomy at 1.5 years old, Santulli stoma revision (NA) with S/C connection at 5 years old
10	Proximal enterostomy (160 cm) at 4 years old
11	Proximal enterostomy (NA) at day 20, BK stoma revision (90 cm) with ileal connection at 3 years old, BK stoma revision (85 cm) with ileal connection at 5 years old, enterostomy (75 cm) with right hemicolectomy at 8 years old
12	Proximal enterostomy (115 cm) at 9 months old, Santulli stoma revision (100 cm) with ileal connection at 2.5 years old, BK stoma revision (100 cm) with T/C connection at 3 years old
13	Proximal enterostomy (50 cm) at 1 month old, enterostomy (30 cm) with distal ileal resection at 2 months old, enterostomy (90 cm) with subtotal colectomy at 8 years old
14	BK stoma revision (NA) with ileal connection at 6 months old, BK stoma revision (100 cm) with T/C connection at 3 years old
15	BK stoma revision (95 cm) with ileal connection at 1 year old, BK stoma revision (55 cm) with T/C connection at 2 years old
16	Santulli stoma revision (30 cm) with ileal connection at 8 months old, Santulli stoma revision (45 cm) with ileal connection at 19 months old, BK stoma revision (65 cm) with ileal connection at 3 years old
17	BK stoma revision (75 cm) with T/C connection at 3 years old
18	BK stoma revision (50 cm) with T/C connection at 10 months old
19	BK stoma revision (40 cm) with ileal connection at 1 year old, BK stoma revision (40 cm) with T/C connection at 2 years old

Supplemental manuscript:

Subsequent non-transplant surgical interventions after the initial enterostomy

The details of subsequent non-transplant surgeries are summarized in Table S1. Pt 1 and Pt 6 underwent tube-enterostomy following multiple intestinal resections. The residual intestinal length and IR were 76 cm and 0.51, respectively, in Pt 1 and 75 cm and 0.58, respectively, in Pt 6. The limited length of the distal colon was retained in Pt 1, and the rectum was connected distally to the intestine in Pt 6. Both patients were unable to eat orally and required a large amount of drainage through the tube-enterostoma, which significantly impaired their quality of life. Both patients underwent iTx at a later stage of life.

Six patients (Pt 2, Pt 3, Pt 8, Pt 10, Pt 11, and Pt 13) retained end-enterostomas after several surgical interventions with small intestinal lengths ranging from 75 to 160 cm (the IRs of these patients were 0.65, 1.03, 1.24, 1.65, 0.60, and 0.86, respectively) at the time of the most recent interventions. None

of the patients utilized a distal colon. Pt 2 and Pt 3 underwent ITx at the ages of 15 and 11 years, respectively, as described in a separate section. Two patients (Pt 8 and Pt 10 [both 12 years old]) were in a stable condition with a drainage tube placed in the end stomas. As the parents of both these patients were reluctant to have their children undergo another surgical intervention, further interventions were not performed. Pt 11 (9 years old) and Pt 13 (11 years old) were not offered stoma revision because the contrast study revealed a microcolon presumably due to disuse atrophy. All patients were dependent on PN support with daily irrigation from the stomas.

Pt 4, Pt 5, and Pt 9 underwent intestinal resection with an antimesenteric 10-cm-long colon patch following initial stoma creation to promote the absorption of intestinal content at the ages of 3, 9, and 1.5 years. Subsequently, they underwent Santulli-type stoma revision utilizing the distal sigmoid colon at the ages of 12, 9, and 5 years, respectively, as illustrated in Figure 1b. Their IRs at the time of Santulli-type stoma revision were 0.81, 1.27, and 0.96, respectively. Initially, anal defecation was observed in all patients after Santulli-type stoma revision; however, they gradually developed difficulty in passing stool from the anus. Pt 4 and Pt 5 required subsequent proximal jejunostomy and gastrostomy because of abdominal distention 6 and 4 years, respectively, after Santulli-type stoma revision.

Eight patients (Pt 7, Pt 12, Pt 14, Pt 15, Pt 16, Pt 17, Pt 18, and Pt 19) underwent BK-type stoma revision following multiple surgical interventions (the mean number of surgeries was 3.87) at ages ranging from 10 months to 12 years old. Pt 7, Pt 12, and Pt 16 underwent Santulli-type stoma revision before BK-type stoma revision. In Pt 7, Pt 14, Pt 16, and Pt 19, the distally anastomosed ileum was utilized in the BK-type stoma revision (Figure 1d), and they developed worse abdominal distension with recurrent severe enteritis; thus, distal ileal resection with right hemicolectomy was performed later for Pt 7, Pt 14, and Pt 19 (Figure 1c). With the current type of stoma (Figure 1c), Pt 12 (10 years old) and Pt 15 (7 years old) were off PN with good oral intake at the time of the study; however, the other six patients were dependent on daily PN to various extents.
